# Pathogen-Specific Immune Fingerprints during Acute Infection: The Diagnostic Potential of Human γδ T-Cells

**DOI:** 10.3389/fimmu.2014.00572

**Published:** 2014-11-13

**Authors:** Matthias Eberl, Ida M. Friberg, Anna Rita Liuzzi, Matt P. Morgan, Nicholas Topley

**Affiliations:** ^1^Cardiff Institute of Infection and Immunity, School of Medicine, Cardiff University, Cardiff, UK; ^2^Cardiff and Vale University Health Board, Cardiff, UK; ^3^Institute of Translation, Innovation, Methodology and Engagement, School of Medicine, Cardiff University, Cardiff, UK

**Keywords:** bacterial infection, point-of-care diagnosis, biomarkers, innate immunity, local inflammation

## Apocalypse Now: The End of Modern Medicine as We Know It

Gentlemen, it is the microbes who will have the last word. [Messieurs, c’est les microbes qui auront le dernier mot]. – Louis Pasteur, 1822–1895

The last 200 years have seen a dramatic reduction in the prevalence and severity of microbial infections, due to the implementation of groundbreaking measures ranging from improved sanitation and hygiene and the introduction of aseptic techniques to the development of successful vaccines and the discovery of effective antibiotics. Devastating infections that were common until the late nineteenth century such as cholera, diphtheria, plague, syphilis, tuberculosis, and typhoid came into the reach of effective control, at least in developed countries, and with a minimized risk of wound infections surgical procedures began to revolutionize modern medicine. Antibiotics, in particular, radically transformed the treatment and prevention of microbial infections and have saved millions of lives since their introduction (1). However, antibiotic usage is invariably linked to the selective pressure it exerts on the target organism to develop escape strategies (2).

We are at present witnessing how the pendulum begins to swing backwards, with anti-microbial resistances developing on an unprecedented global scale. New classes of Gram-positive and Gram-negative “superbugs” are emerging and spreading at an alarming rate, some of which are virtually insusceptible to all available drugs (3–5). The once apocalyptic vision of a “post-antibiotic era” where common infections and minor injuries may result untreatable and eventually fatal is rapidly becoming a real possibility (1, 2, 6, 7), heralding what Margaret Chan, Director-General of the WHO, in 2012 called “the end of modern medicine as we know it.” The appearance of multidrug-resistant bacteria has been identified by the WHO, the Centers for Disease Control and Prevention (CDC) in the USA and their European counterpart, the ECDC, as one of the major global health challenges humankind is facing in the twenty-first century (8–10). According to Sally Davies, the UK Chief Medical Officer, “there are few public health issues of greater importance than anti-microbial resistance in terms of impact on society” (11).

There is now an urgent call for anti-microbial stewardship programs that aim to prescribe antibiotics more prudently, and to tailor their use to defined patient groups who will benefit most. The fact that the prevalence of resistance appears to correlate directly with antibiotic consumption across different countries (12) argues in favor of the immediate effectiveness of such tightly controlled programs. As highlighted in a recent Outlook issue in *Nature*, “the potential to save lives with faster and more targeted diagnoses, decrease unnecessary and often incorrect prescriptions, and even help identify early on where bacterial resistance could occur, will have a drastic effect on the way patients are treated” (13).

## Mission Impossible: The Fundamental Flaws of Conventional Diagnosis

When it concerns the search for pathogenic organisms suspected in the diseased body, in the first instance bacteria, then during conventional microscopic examination carried out without special preparations and artifices one encounters the most substantial, at times virtually insurmountable, obstacles. [Wenn es sich nun darum handelt, die im erkrankten Körper vermutheten pathogenen Organismen, zunächst Bacterien, aufzusuchen, so begegnet man bei der gewöhnlichen ohne besondere Vorbereitungen und Kunstgriffe ausgeführten mikroskopischen Untersuchung den erheblichsten, stellenweise geradezu unübersteiglichen Hindernissen]. – Robert Koch, 1843–1910 (14)

More than a century after Robert Koch’s landmark discovery of the causative agents of anthrax, cholera, and tuberculosis, the diagnosis of suspected infections still depends largely on the definitive identification of the likely pathogen in biological samples. However, standard microbiological culture is inefficient and slow (typically >1–2 days, for a confirmed diagnosis of tuberculosis >4 weeks), and in many cases no organism can be grown despite clinical signs of infection, indicating that conventional diagnostic methods are not specific and/or rapid enough to target therapy (15–17). Early management of patients with acute symptoms who require immediate medical intervention, including virtually all hospital-based infections, thus remains largely empirical. As direct consequence, the fundamental uncertainty about the real cause underlying the clinical signs observed leads to inappropriate and unnecessary treatments exposing patients to drug-related side effects; raising the risk of opportunistic, chronic, or recurrent infections; and contributing to the emergence and spread of multidrug resistance (1–7). This dilemma eventually results in potentially avoidable patient morbidity/mortality, and imposes a considerable burden on health care systems and societies (8–11). There remains an unmet clinical need for rapid and accurate diagnostic tests for patients with acute infections. According to Kessel and Sharland (18), “new technology focusing on rapid diagnosis of specific bacteria and resistance genes, along with combination biomarkers indicating bacterial or viral infections, especially if adapted to near patient testing, could have a major impact on targeting appropriate antibiotic treatment.”

In order to circumvent the almost insurmountable obstacles of a rapid and accurate identification of the causative pathogen by traditional microbiological techniques, efforts are being made to utilize state-of-the-art molecular methods. Approaches based on the detection of microbial nucleic acids, cell wall constituents, or other unique features of distinct pathogens by PCR, chromatography, or mass spectrometry certainly complement culture-based tests and speed up microbial identification, yet they require considerable resources and may not be applicable to primary care or home settings (19–23). Moreover, they do not provide information about the pathogenicity of the identified species and its interaction with the host. Of note, neither microbiological nor molecular methods discriminate between pathogens causing disease, asymptomatic carriage, and sample contaminants, and thus even positive test results require extensive interpretation by the treating physician (24–26).

There is a plethora of disease-related markers that are commonly assessed by clinicians to aid a correct diagnosis, ranging from basic blood and urine parameters to indicators of tissue damage, tumor progression and autoimmunity, among others. However, there is a conspicuous paucity of biomarkers for accurate diagnosis of microbial disease. Current biomarkers of inflammation such as C-reactive protein (CRP) or procalcitonin (PCT) are often not sensitive or specific enough and are only poor surrogates for acute infections (22, 27, 28). The vast majority of research on novel diagnostics has so far focused on identifying individual factors and assessing their performance in isolation. Yet, it may come as no surprise that none of these proposed parameters have reached sufficient discriminatory power on their own, given the complex and multifactorial processes underlying local and systemic inflammatory responses to a broad range of pathogens (29, 30). As a result, neither the direct identification of the causative pathogen nor the measurement of currently used biomarkers of inflammation is sufficiently accurate or rapid for a reliable point-of-care diagnosis of acute microbial infection.

## Quantum of Solace: Exploitation of Pathogen-Specific Host Responses for Novel Diagnostics

The immune system appears to have originated as a set of effector cells having multiple distinct receptors that discriminate self from infectious non-self by recognition of patterns found exclusively on microorganisms. – Charles A. Janeway, Jr., 1943–2003 (31)

Key to developing better and stratified approaches to treating infection is a detailed understanding of the intricate host–pathogen relationships in disease, in order to exploit the unique sophistication of the human immune system for diagnostic and therapeutic purposes (32, 33). In a radical departure from current practice, our research is based upon the premise that each type of infection evokes a distinct pathogen-specific host response – what we refer to as “immune fingerprint.” A patient’s early anti-microbial response itself is likely to provide far more detailed insight into the true cause and severity of acute infections than conventional methods, independently of the subsequent clinical course of the disease (34). The human immune system is a highly complex network of interdependent cellular and humoral players that has evolved over millions of years in order to survey the body for potentially hazardous structures and initiate an appropriate defense. The communication with invading micro-organisms thus occurs at multiple levels, giving rise to a plethora of biomarkers of potential relevance for diagnostic purposes. Different pathogens interact uniquely with different components of the innate immune system due to the efficient self/non-self discrimination based on conserved microbial signals such as non-methylated bacterial DNA, bacterial flagella, and cell wall constituents. These structures are typically recognized by members of the Toll-like receptor family and/or other pattern recognition receptors expressed by sentinel cells (35–37). However, there is also emerging evidence that certain types of innate or “unconventional” T-cells such as γδ T-cells and mucosal-associated invariant T (MAIT) cells are able to detect common microbial metabolites through their T-cell receptors, by sensing intermediates of the non-mevalonate and riboflavin biosynthesis pathways that are unique to certain types of microorganisms (38, 39).

Vγ9/Vδ2 T-cells represent a unique subpopulation of human T-cells (40, 41) that appears to have a particularly crucial role in contributing to immune fingerprints of diagnostic relevance (34). This is due to their exquisite responsiveness to the microbial isoprenoid precursor (*E*)-4-hydroxy-3-methyl-but-2-enyl pyrophosphate (HMB-PP) that is produced by the majority of Gram-negative pathogens and a large proportion of Gram-positive species such as *Clostridium difficile, Listeria monocytogenes*, and *Mycobacterium tuberculosis*, while it is not found in other bacteria including staphylococci and streptococci as well as fungi (42–44). The rapid and sensitive response of Vγ9/Vδ2 T-cells to a broad range of pathogens evokes Janeway’s criteria for a “pathogen-associated molecular pattern” in that HMB-PP is an invariant metabolite in many different species that is essential in the microbial physiology but absent from the human host (43, 45). Bacterial extracts prepared from HMB-PP producing species typically activate Vγ9/Vδ2 T-cells much stronger than extracts prepared from HMB-PP deficient micro-organisms (42, 44, 46), and peripheral and/or local Vγ9/Vδ2 T-cell levels are often elevated in patients infected with defined HMB-PP producing pathogens (43, 47). Elegant proof of concept for this responsiveness comes from the demonstration that HMB-PP producing wildtype *L. monocytogenes* activate Vγ9/Vδ2 T-cells far better, both *in vitro* (48) and in primate models *in vivo* (49), than genetically engineered *L. monocytogenes* that are identical to the parental strain except for an inability to produce HMB-PP. Similarly, overexpression of HMB-PP synthase through genetic manipulation increases the stimulatory potential of bacteria such as *E. coli, L. monocytogenes, M. tuberculosis*, and *Salmonella enterica* on Vγ9/Vδ2 T-cells *in vitro* (42, 46, 48, 50, 51) and *in vivo* (52). Our own data demonstrate that even in heterogeneous patient cohorts infected with a whole spectrum of diverse bacteria, differences in Vγ9/Vδ2 T-cell frequencies between patients with microbiologically confirmed infections caused by HMB-PP producing and HMB-PP deficient species remain apparent. This is true both for peritoneal dialysis patients with acute peritonitis as an exemplar of localized immune responses restricted to the peritoneal cavity (34, 46, 53), as well as on a systemic level in the peripheral blood of critically ill patients with severe sepsis (54). Most importantly, studies in patients with acute peritonitis suggest that a diagnostic test measuring local Vγ9/Vδ2 T-cells on the first day of presentation with acute symptoms may not only indicate the presence of Gram-negative (predominantly HMB-PP producing) bacteria but also identify patients at an increased risk of inflammation-related downstream complications (34).

**Figure 1:**
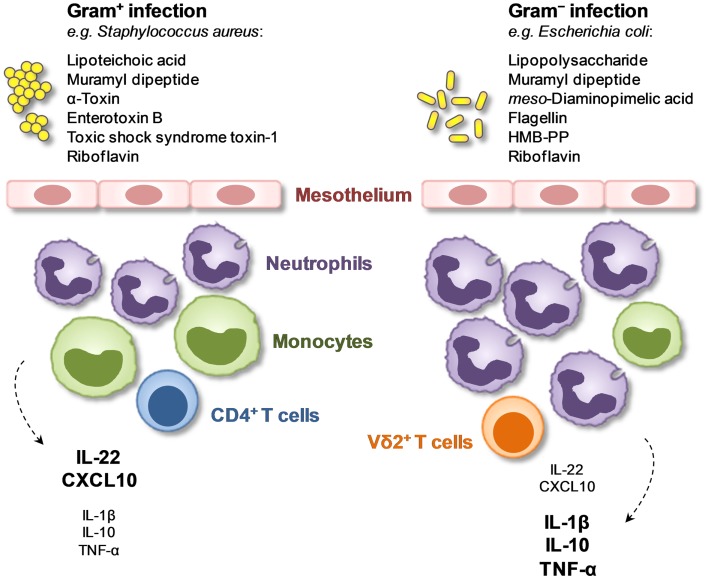
**Local immune fingerprints in peritoneal dialysis patients on the day of presentation with acute peritonitis**. Shown are cellular and humoral biomarkers that are associated with the presence of Gram-positive or Gram-negative bacteria and that may be exploited for novel diagnostic tests (34).

The exquisite responsiveness of Vγ9/Vδ2 T-cells and other unconventional T-cells to microbial metabolites shared by certain pathogens but not by others identifies these cell types as key constituent of diagnostically relevant immune fingerprints at the point of care. This is especially the case when Vγ9/Vδ2 T-cell levels are assessed locally and when they are combined with other powerful discriminators such as peritoneal proportions of neutrophils, monocytes, and CD4^+^ T-cells in the inflammatory infiltrate as well as intraperitoneal concentrations of certain soluble immune mediators (34) (Figure 1). Such a combination with further parameters provides additional information as to the precise nature of the causative pathogen, for instance to distinguish between immune responses induced by Gram-negative (LPS producing) and Gram-positive (LPS deficient) bacteria, and is also likely to help increase sensitivity owing to the age and gender-dependent variability of Vγ9/Vδ2 T-cell levels (55). Pathogen-specific immune fingerprints that discriminate between certain subgroups of patients (e.g., with Gram-negative vs. Gram-positive bacterial infections) can be determined within hours of presentation with acute symptoms, long before traditional culture results become available, and by guiding early patient management and optimizing targeted treatment will contribute to improving outcomes and advancing antibiotic stewardship. It remains to be investigated how much these findings on diagnostic immune fingerprints in peritoneal dialysis patients can be extended to other local or systemic scenarios to diagnose infections at the point of care, and whether they can also be applied to monitoring the course of the disease and the response to treatment.

Applied research on γδ T-cells has so far focused predominantly on their use for novel immunotherapies against different types of cancers (56–58). Thirty years after the unexpected cloning of the TCRγ chain (59, 60) and 20 years after the first description of microbial “phosphoantigens” as specific activators of human Vγ9/Vδ2 T-cells (61, 62), the diagnostic potential of γδ T-cells is only beginning to unfold (34, 47, 63, 64).

## Conflict of Interest Statement

The authors declare that the research was conducted in the absence of any commercial or financial relationships that could be construed as a potential conflict of interest. The Specialty Chief Editor Bernhard Moser declares that, despite being affiliated to the same department as authors Matthias Eberl, Ida M. Friberg, Anna Rita Liuzzi, Matt P. Morgan and being affiliated to the same institution as Nicholas Topley, and despite having collaborated on publications in the last 2 years with Matthias Eberl, Anna Rita Liuzzi, Matt P. Morgan and Nicholas Topley, the review process was handled objectively.

## Abbreviation

HMB-PP, (*E*)-4-hydroxy-3-methyl-but-2-enyl pyrophosphate.
